# Targeting primary mediastinal B-cell lymphoma

**DOI:** 10.18632/oncotarget.2351

**Published:** 2014-08-14

**Authors:** Simon D. Wagner

**Affiliations:** Department of Cancer Studies and Molecular Medicine and MRC Toxicology Unit, University of Leicester, Lancaster Road, Leicester, UK

Primary mediastinal B-cell lymphoma (PMBL) is a high grade B-cell lymphoma with distinct clinical and molecular features, which is confined to the mediastinum at presentation although relapsed disease can be more widespread. PMBL accounts for just 3 % of all non-Hodgkin's lymphomas and tends to present in young women. It is conventionally treated with combination chemotherapy, usually followed by radiotherapy. The overall survival obtained with these regimens is 70 to 80 *%* but there may be significant risks of secondary malignancies and cardiac problems due specifically to the radiotherapy component of treatment, which has prompted attempts to reduce these problems with dose-adjusted regimens[1].

PMBL is believed to derive from thymic B-cells and may share this origin with nodular sclerosis classical Hodgkin's lymphoma with which it has some pathological and genetic features in common. Both conditions show amplification of the JAK2 locus on chromosome 9. Mutations in SOCS1 may co-operate with over-expression of JAK2 to drive STAT6 signalling in PMBL. Interestingly, the STAT6 DNA binding domain is mutated in 36 % of cases suggesting altered function of this transcription factor although the implications of this finding remain to be elucidated. In PMBL cell lines JAK2 exerts its effects not only through STAT6 but also directly through phosphorylation of histone H3 at tyrosine 41 (H3Y41). The effects of this epigenetic modification appear to be activation of MYC and its transcriptional programme suggesting a direct mechanism leading to proliferation [2]. Another transcription factor, BCL6, is also highly expressed in PMBL. BCL6 is essential for normal germinal centre B- and T-cells and the generation of high affinity antibodies. Experimental evidence demonstrates that BCL6 drives lymphoid hyperplasia and possibly ultimately lymphomagenesis in mouse thymic B-cells [3] suggesting that it may have a specific role in the development of PMBL.

The BCL6 consensus binding sequence is similar to the binding sequences of the STAT family of transcription factors [4] and this has suggested that BCL6 modifies STAT signalling through direct competition for binding. The converse may also be true and STATs can regulate BCL6 expression although this is more controversial with STAT5 inducing BCL6 in primary cells whilst STAT5 and STAT6 repress in lymphoma cell lines.

Collectively, in this rare and difficult-to-study disease, BCL6 and JAK2-STAT6 have very prominent roles in driving proliferation. BCL6 accomplishes transcriptional repression by binding NCOR1/NCOR2/ BCOR co-repressors, which in turn recruit histone. Small molecule inhibitors of BCL6 have been produced and shown to be effective in preclinical models of diffuse large B-cell lymphoma [5]. JAK2 inhibitors are more developed and have been employed in clinical trials for the treatment of certain myelo-proliferative conditions. An additive effect in causing apoptosis was obtained utilising the BCL6 inhibitor, 79-6, and the JAK2 inhibitor, TG101348 [6]. Although this is a promising preclinical result it is likely that these novel small molecule inhibitors will need to be employed in conjunction with conventional chemotherapy. The standard regimen R-CHOP contains rituximab, cyclophosphamide, hydroxydaunorubicin, vincristine and prednisolone. Now Häberle et al. [7] have shown that siRNA mediated knockdown of BCL6 alone causes loss of viability of PMBL cell lines. Knockdown of STAT6 alone did not produce such pronounced effects but when some of the components of R-CHOP (the anthracycline antibiotic, doxorubicin, the anti-CD20 monoclonal antibody, rituximab and the microtubule poison, vincristine) were included, killing was enhanced. There is clearly a differential effect of these combinations in the three different cell lines used in the study possibly reflecting important heterogeneity in response in PMBL, but Häberle et al. have shown a methodology for assessing combinations of novel agents and demonstrate how new agents could be combined with old to produce treatments that are potentially as effective but lack the myelotoxicity of current regimens.
